# The practices of isolating tuberculosis infectious patients at hospitals of Vhembe district, Limpopo Province

**DOI:** 10.4102/phcfm.v6i1.555

**Published:** 2014-11-10

**Authors:** Takalani G. Tshitangano

**Affiliations:** 1 Department of Public Health, University of Venda, South Africa

## Abstract

**Background:**

Airborne infections pose a serious threat to susceptible individuals whenever they are together in confined spaces with patients coughing up tuberculosis (TB) bacilli. In healthcare facilities, those with infectious TB should, as far as possible, be isolated from non-infectious patients in order to prevent exposure to the infectious droplet nuclei generated by infected patients.

**Aim:**

This article aims to describe the use of masks and isolation of infectious TB patients at hospitals of Vhembe district, Limpopo Province in order to inform future policy and practices.

**Setting:**

This study was conducted at seven of the eight hospitals in Vhembe district.

**Methods:**

A cross-sectional qualitative design of a descriptive nature was used. Purposive sampling was used to select 57 focus group participants. Necessary approval, permission and clearance were obtained. The participants’ rights were respected.

**Results:**

This study confirmed that TB cubicles were not reserved for patients with infectious TB and that many TB inpatients at hospitals of Vhembe district were not isolated; masks were not used consistently or appropriately by patients, staff or visitors. Furthermore, the movement of TB inpatients in isolation was not restricted.

**Conclusions:**

There is an unnecessary risk of becoming infected with TB at the rural hospitals of Vhembe district as a result of incorrect isolation practices. The development and implementation of a quality control programme, as well as ongoing training at the hospital level, would improve the TB infection control measures practised by healthcare workers at hospitals in Vhembe district and reduce the risk of acquiring TB at these hospitals.

## Abstrait

**L'habitude d'isoler les patients infectés par la tuberculose dans les hôpitaux du district de Vhembe, dans la province du Limpopo**.

**Contexte:**

Les infections aéroportées sont une sérieuse menace pour les personnes sensibles quand elles se trouvent ensemble dans un espace confiné avec des patients qui toussent et projettent des bacilles de tuberculose (TB). Dans les établissements de santé, les personnes infectées par la tuberculose devraient, autant que possible, être isolées des patients non-infectieux pour éviter l'exposition aux noyaux de gouttelettes infectieuses produites par les patients infectés.

**Objectif:**

Cet article a pour objet de décrire l'utilisation de masques et l'isolation des patients atteints de tuberculose infectieuse dans les hôpitaux du district de Vhembe, dans la province du Limpopo, afin d’élaborer les politiques et pratiques futures.

**Cadre:**

Cette étude a été menée dans sept des huit hôpitaux du district de Vhembe.

**Méthodes:**

On a utilisé une étude transversale qualitative de nature descriptive. On a utilisé l’échantillonnage au jugé pour choisir 57 participants pour le groupe témoin. On a obtenu l'approbation, la permission et l'autorisation nécessaires. On a respecté les droits des participants.

**Résultats:**

Cette étude a confirmé que les compartiments pour tuberculeux n’était pas réservés aux patients atteints de tuberculeuse infectieuse et que beaucoup de patients tuberculeux hospitalisés dans les hôpitaux du district de Vhembe n’étaient pas isolés; les masques n’étaient pas utilisés systématiquement ou correctement par les patients, le personnel ou les visiteurs. En outre, les mouvements des patients tuberculeux hospitalisés en isolation n’étaient pas limités.

**Conclusions:**

Il y a des risques inutiles d’être infecté par la tuberculose dans les hôpitaux ruraux du district de Vhembe en raison des mauvaises pratiques d'isolation. Le développement et la mise en œuvre du programme de contrôle de qualité, ainsi qu'une formation continue au niveau de l'hôpital, améliorerait les mesures de contrôle de l'infection tuberculeuse pratiquées par le personnel hospitalier dans les hôpitaux du district de Vhembe et réduirait les risques de contagion tuberculeuse à ces hôpitaux.

## Introduction

Airborne infections pose a serious threat to susceptible individuals whenever they are placed together with the index case in confined spaces.^1^ According to Escombe et al., airborne tuberculosis (TB) transmission occurs particularly in overcrowded settings containing TB infectious patients, such as hospitals, clinics, homeless shelters, and prisons.^1^ Therefore, the World Health Organization (WHO) advises that infectious TB patients be isolated from other patients in order to protect these other patients from the infectious droplet nuclei that infectious patients generate.^2^

The US Centers for Disease Control and Prevention (CDC) describes isolation as ‘the separation of persons who have a specific infectious illness from those who are healthy and the restriction of their movement to stop the spread of that disease’.^3^ Sissolack, Marais and Mehtar see quarantine as another form of isolation.^4^ However, quarantine is described by the CDC as the ‘separation and restriction of movement of persons who, while not yet ill, have been exposed to an infectious agent and therefore may become infectious to stop the spread of an infectious disease’.^3^ The WHO suggests a third less effective way of separating infectious TB patients from non-infectious patients, namely, to establish a separate area within a hospital ward.^2^ In this study, isolation refers to separation of individuals having a particular disease or who have been exposed to an infectious agent from those who are healthy; this includes the establishment of a separate area within a hospital ward and the restriction of the movement of the infected patients in order to minimise the spread of disease.

The problem is that, according to the WHO, ‘of every 100 hospitalized patients at any given time, 7 in developed and 10 in developing countries will acquire at least one of the infections acquired in health care settings’.^5^ Tuberculosis is one of these infections. Authors worldwide warn that the risk of becoming infected with TB in healthcare settings is increasing daily.^2, 3, 4, 5^ Lin et al. indicated that infections acquired in healthcare settings represent an important indicator of healthcare quality and patient safety, which makes it an important health issue for research.^6^ The WHO acknowledges that infections acquired in healthcare settings are a recognised public health problem and a global public threat for both patients and healthcare professionals.^7^ Mixing infectious TB patients with patients who do not have TB, especially those with HIV or other immune-compromised conditions, fuels TB transmission risks.^5^

A comprehensive infection, prevention and control (IPC) programme helps to decrease the spread of TB, including drug-resistant TB. This study was part of a larger study that was conducted in 2012, which examined other IPC interventions with regard to TB, namely, availability of a TB infection control plan at hospitals, existence of health service delay in TB diagnosis and treatment, TB infection control trainings for healthcare workers (HCWs), environmental control practices and sputum collection practices.^8, 9^ These studies found that IPC interventions were not aligned to the WHO policy on TB infection control in healthcare facilities, households and congregate settings, because of a lack of TB infection control plans in hospitals.^10^

## Purpose of the study

This article aims to describe the use of masks and isolation of infectious TB patients at hospitals of Vhembe district, Limpopo Province in order to inform future policy and practice.

## Objectives

The objectives of this study were as follows:

- Explore and describe how the movement of an infectious TB patient was handled in the isolation ward.
- Explore and describe the management of visitation in the isolation ward.
- Explore and describe where infectious TB patients were placed in the hospital.
- Explore and describe the use of masks in the TB ward or isolation cubicles at hospitals in Vhembe district.

## Research methods and design

### Study design

In this study, a cross-sectional qualitative design of a descriptive nature was used.^11^ Vhembe district has eight public hospitals. One hospital, however, is a psychiatric hospital and does not admit TB patients. This study was thus conducted in 2012 at only seven of the eight hospitals of Vhembe district. The target population of this study was all the HCWs at these hospitals. The HCWs in this study were professionals involved in the care of TB patients, namely, laboratory staff members, surgical ward nurses, antiretroviral (ARV) clinic nurses, TB focal point staff, paediatric ward nurses, outpatient department (OPD)/casualty nurses, X-ray staff members, TB ward nurses, medical ward nurses, infection control nurses, occupational health and safety (OHS) nurses, pharmacy staff members, sub-acute ward nurses, maternity ward nurses, psychiatric ward nurses and doctors. This target population was chosen because HCWs are in direct contact with infectious patients and are more at risk of acquiring infectious conditions such as TB.^6^

### Study population and sampling strategy

Purposive sampling of a maximum variation type^12^ was used to select a variety of representative focus group participants believed to have the necessary TB control knowledge needed in this study. The participants in the focus group discussions varied, comprising the deputy manager of nursing, laboratory staff members, surgical ward nurses, ARV clinic nurses, TB focal point staff, paediatric ward nurses, OPD/casualty nurses, X-ray staff members, TB ward nurses, medical ward nurses, infection control nurses, OHS nurses, pharmacy staff members, sub-acute ward nurses, maternity ward nurses and psychiatric ward nurses. Although doctors were amongst the population targeted for this study, they did not consent to participation and thus did not take part in the study.

The sample size for the focus group discussions was one focus group per participating hospital, making up 7 focus groups. Each focus group comprised five to 10 members, making a total sample size of 57 participants.

The central question for the focus group discussions was: ‘How would you describe the practices of isolating an infectious TB patient in this hospital?’ In response to the answers given by the focus group members, follow-up probing questions were asked, as prepared for in the unstructured focus group discussion guide. The content and construct validity of the focus group discussion guide and checklist were ensured through consultation with the Vhembe district TB coordinator as well as the WHO TB infection control policy.^10^

### Data collection

The pilot study at the first hospital afforded the researcher the opportunity to pre-test the focus group discussion guide as well as the checklist in order to check the reliability of the checklist by using a test–re-test and an inter-rater test, in order to determine whether the wording and constructs were clear and to evaluate the feasibility of the entire study. The data collection tools and processes were then readjusted accordingly. The data collected from the pilot study formed part of the empirical data for this study so as not to lose important information.

A checklist was developed to record participants’ responses to the central question that asked: ‘Is there a TB ward away from non-infectious wards in this hospital’. This checklist had 3 columns, two for answers of ‘Yes’ and ‘No’ and a further column that allowed the participants to describe the availability of TB wards that are kept separate from the non-infectious wards.

### Data analysis

Mixed methods were used to analyse the data. Qualitative data collected through focus group discussions were analysed using the open coding method following Tesch's^13^ 8-step criteria as described by Creswell,^14^ whereas data collected through the checklist were analysed using quasi-statistics, where the number of hospitals with or without a separate TB ward were counted.^11, 12^ The results from quasi-statistics as well as those from open coding analysis were combined, giving rise to the conclusions of the study. Lincoln and Guba's^15^ model comprising four criteria or measures to ensure trustworthiness of the study findings, namely, truth value (credibility), applicability (transferability), consistency (dependability) and neutrality (confirmability), was adopted.

### Trustworthiness

In order to ensure credibility of the study findings, three data-collection instruments (focus group discussions; document study; and observation) presented methodological triangulation, whilst the use of both quantitative and qualitative data analysis methods demonstrated data analysis triangulation. The researcher also took photographs of what was observed for further reference. Member checks afforded participants an opportunity to verify whether the researcher had captured exactly what they had said. Prolonged engagement of participants during focus group discussions, which lasted one to one-and-a-half hours, enabled the researcher to collect all the information regarding TB infection control practices until data saturation was achieved.^15^ Persistent observations which lasted from one-and-a-half to two hours per hospital, helped the researcher to gather information regarding all environmental TB control measures practised in the study hospitals.

In order to ensure transferability of the study findings, a thick description was documented in clear simple language regarding how themes were identified, how code books were built, how subcategories were induced and how categories emerged, so as to afford readers a chance to decide for themselves if the results are transferable to their own contexts.

In order to ensure dependability of the study findings, the auditor (who happened to be a neutral colleague from the same university) examined the running account of the process of inquiry as well as the findings and recommendations, attesting to the fact that the findings are supported by the data and that the study is internally coherent. An extensive and detailed record of the study process was documented for others to replicate.^11^ The proposal for this study was presented to a panel of experts (comprising professors and doctors) serving on the Higher Degrees Ethics Committee of the University of Venda; and a certificate of ethical clearance was issued by the university as a way of showing its belief in the methodology's ability to yield dependable results. The proposal was also evaluated by the Private Research Ethics Committee of the University of Limpopo, who issued approval to conduct the study, again as a way of showing a belief in the ability of the methodology to yield dependable results. The proposal for this study was presented at the International Nurses’ Conference held from 27 June to 4 July 2009 in Durban, South Africa for peer-review and the inputs from this conference were taken into consideration. The researcher is an experienced healthcare professional in the field of TB management. The researcher's TB management knowledge and expertise added value to the process of data collection, coding, formulation and naming of subcategories, establishment of relationships and model development, which attest to the dependability of the findings.

In order to ensure confirmability of the study findings, the researcher has thus far published 11 papers in Department of Higher Education and Training-accredited peer-reviewed journals. Three of the published papers have been presented at national conferences for peer-review purposes.

### Ethical considerations

Approval for the study was obtained from both the University of Venda (UNIVEN) Higher Degree Committees and the Limpopo Provincial Department of Health. Ethical clearance was obtained from the UNIVEN Ethics Committee (project number SHS/10/PDC/02) and the Limpopo Provincial Department of Health provided a letter that gave permission for data collection at each of the seven hospitals. Ethical clearance obtained from UNIVEN Ethics Committee, together with the permission letter from the province, were used to negotiate entrance to each hospital where data were collected. Informed written consent was obtained from each participant. Ethical principles such as the principle of justice, beneficence and self-determination were observed when dealing with participants. In addition, hospitals where data was collected were allocated codes A–G to ensure that the participants’ rights to privacy, anonymity and confidentiality were respected.

## Results

This study confirmed that TB cubicles were not reserved for patients with infectious TB and that many TB inpatients at hospitals of Vhembe district were not isolated; masks were not used consistently or appropriately by patients or staff or visitors. Furthermore, the movement of TB inpatients in isolation was not restricted. These findings were arranged into categories, which are discussed in detail hereunder. Table 1 provides details of the participants whose views have been quoted in the text.

**TABLE 1:** Participants’ demographics.

Participant number	Position	Hospital	Gender	Age
1	Medical ward nurse	D	F	48
2	General ward nurse	G	F	33
3	Medical ward nurse	B	F	37
4	TB ward nurse	C	M	52
5	Male medical ward nurse	C	M	46
6	Female medical ward nurse	C	F	43
7	Female medical ward nurse	F	F	46
8	Male medical ward nurse	F	F	43
9	TB ward nurse	A	F	47

TB, tuberculosis.

### Tuberculosis inpatients were not isolated; cubicles were not reserved

The probing question was: ‘Is there a TB ward away from the non-infectious wards in this hospital?’ Four hospitals did not have dedicated TB wards, but had TB cubicles established within the medical ward. Only two of the hospitals had TB wards established a distance away from the other wards, but these wards were used in different ways depending on the hospital. One hospital used the TB ward to isolate only TB inpatients who were on streptomycin. The other hospital had divided its TB ward into cubicles, one for multi-drug-resistant (MDR)-TB inpatients; one for non-pulmonary TB inpatients; one for sputum-negative TB inpatients; one for sputum positive inpatients; and the other one for TB suspect inpatients. One hospital did not have an area or ward dedicated for either TB suspects or confirmed TB inpatients. In this hospital, infectious and non-infectious inpatients were mixed together in a medical ward (see Table 2).

**TABLE 2:** Availability of tuberculosis wards away from non-infectious wards: Quantitative data.

Hospital	Is there a tuberculosis ward away from non-infectious wards in this hospital?
**A**	**Yes:** There is a cubicle for TB suspects in the TB ward; TB ward is for all confirmed TB cases.
**B**	**No:** TB patients are nursed in medical wards
**C**	**No:** Separate areas for TB suspects for now. TB patients are nursed in a medical ward. TB ward is for patients on streptomycin only.
**D**	**Yes:** TB suspects are nursed in TB ward in a suspect cubicle. Confirmed cases are nursed in TB wards in their own cubicles.
**E**	**No:** TB patients are admitted in their isolation ward, which isolates all infectious cases, even when non-TB.
**F**	**No:** There is no TB ward. TB patients are nursed in a medical ward, but there are cubicles for TB suspects, one for sputum-positive TB and one for MDR-TB.
**G**	**No:** There is no TB ward. All TB patients are nursed in medical ward amongst other non-infectious patients.

**Conclusion:**	Four hospitals do not have separate TB wards. Of the three that do have TB wards, one utilises this ward only to care for patients on streptomycin.

TB, tuberculosis; MDR, multi-drug resistant.

### The movement of tuberculosis inpatients in isolation was not restricted

When participants were asked how the movement of infectious TB patients was handled in the isolation ward at each hospital, participants from the majority of the hospitals (*n* = 6) indicated that TB inpatient movement was not restricted.

According to one participant from hospital D: ‘Patients’ movement [*sic*] are not restricted’ (P1).

The general ward nurse from hospital G confirmed this, saying: ‘TB patients’ movements are not restricted’ (P2).

A medical ward nurse from hospital B added: ‘TB patients are allowed to go wherever they want’ (P3).

The TB ward nurse, Male medical ward nurse and Female medical ward nurse from hospital C further said that movements of TB patients are not restricted in the hospital ‘because it is difficult to control the movement of a patient who can walk’ (P4, 5, 6).

Only one participating hospital restricted its TB patients’ movements. According to participants 7 and 8 from hospital F, there is a fence around the TB ward which restricts the movement of TB patients. Patients are only allowed to go out when it is necessary and then the appropriate precautionary measures are put in place.

### Masks were not used consistently or appropriately

When a probing question was asked, namely: ‘How would you describe the use of masks in TB ward or isolation cubicles in this hospital?’, participants’ responses included mask use by TB inpatients, visitors and HCWs.

#### Mask use by inpatients

With regard to the use of masks by TB inpatients, the study revealed a variation of mask use, with only two hospitals giving patients surgical masks. One participant, a medical ward nurse from hospital D said: ‘All patients in the TB cubicle put on surgical masks’ (P1).

One hospital was giving patients N95 respirators. Three participants from hospital C said: ‘TB suspects are given N95 mask [*sic*]’ (P4, 5, 6).

That being said, the majority (*n* = 5) of the hospitals did not give patients masks at all. According to the general ward nurse from hospital G: ‘[*I*]solated TB patients are not given disposable masks to put on’ (P2).

This claim was confirmed by participants 7 and 8 from hospital F. Additional confirmation was made by the medical ward nurse from hospital B, who said: ‘[*I*]solated TB patients are not given masks to put on’ (P3).

The researcher further observed that neither patients nor HCWs had masks on in a TB cubicle which is within one of the medical wards in hospital B.

#### Mask use by visitors

With regard to the use of masks by visitors at TB cubicles and wards, participants from the majority of the hospitals (*n* = 6) indicated that visitors are given masks and allowed to see their patients in bed. Out of these six, five gave surgical masks and one gave N95 respirators. One hospital did not give masks at all and yet allowed visitors to see their patients in bed. According to one of the participants from hospital F: ‘Visitors are also given a mask N95. There is a courtesy nurse who supplies visitors with masks and educates them on proper use and disposal’ (P7).

A participants from hospital B said: ‘Visitors see their TB patients in beds. There is no visitor restriction. Masks are sometimes used by visitors’ (P3).

Participants 4, 5 and 6 from hospital C confirmed this by saying that visitors are not restricted and are allowed to see their TB suspect patient without a mask. At the female medical ward, visitors of MDR-TB patients are given masks and are restricted. In the TB ward, visitors are given masks if they are available and are not allowed to see their patients in bed, but rather see them outside in the open air.

A participant from hospital A added:

‘Visitors see their TB suspect or confirmed TB patients in bed. There is no visitor restriction. Visitors get into the ward with paper disposable mask on they take from a container mounted on the wall at the entrance of the TB ward. These masks are disposed into the dirty bin upon leaving the TB wards.’ (P9)

#### Mask use by healthcare workers

With regard to the use of masks by HCWs at TB cubicles and wards, the same confusion regarding which mask to use was revealed by the fact that HCWs at two hospitals put on N95 masks in TB cubicles and wards. According to participants 7 and 8 from hospital F, ‘whenever a nurse enters one of the TB cubicles’ he or she puts on an N95 mask.

Three participants from hospital C said: ‘Nurses put on surgical masks in TB cubicles’ (P4, 5 & 6).

However, HCWs at the majority of the hospitals (*n* = 4) did not put on masks at all in TB cubicles. According to a participants from hospital B: ‘Nurses do not put on masks in TB cubicles’ (P3).

## Discussion

This study discovered that TB inpatients were not isolated and that the TB cubicles were not reserved for patients with infectious TB. Furthermore, the movement of TB inpatients in isolation was not restricted and masks were not used either consistently or appropriately by patients, staff or visitors in wards that cared for infectious TB patients.

These findings concur with those of Basu, Andrew and Poolman, who assert that the ‘capacity for safe airborne isolation does not exist in South African hospitals’.^16^ According to Basu et al., non-XDR (extensively drug resistant)-TB patients are frequently admitted to the wards with other patients who have a TB disease and risk super infection and/or nosocomial infection.^16^ Similarly, Sissolak et al. discovered a total lack of isolation facilities in South Africa.^4^ Knirsch et al. also found that practices and facilities in South Africa were inadequate for both the recognition and isolation of potential TB patients.^17^ Scablon et al. warn that hospitals can be breeding grounds for drug-resistant TB.^18^ Hussey, director of the Institute of Infectious Diseases and Molecular Medicine at the University of Cape Town confirmed that ‘hospitals could be dangerous environments, especially for patients with HIV-compromised immune systems’.^19^ To substantiate that fact, the Church of Scotland hospital study in 2006 discovered that the majority of drug-resistant TB cases were associated with nosocomial infections.^19^ To make matters worse, Port Elizabeth's Joseph Pearson TB hospital nurses reported that ‘MDR-TB patients were contracting XDR-TB strains at an intense rate in a situation where XDR-TB patients were in a different ward from MDR-TB patients’.^4^

Siegel et al. believe that minimising the spread of tuberculosis pathogens in hospitals requires the use of TB isolation wards.^20^ Amon, Girard and Keshavjee concur, stating that ‘under South African law, authorities may detain an individual suffering from an infectious disease until the disease ceases to present a public health risk’,^21^ and state further that the draft government policy guideline^22^ calls for the isolation of all MDR- and XDR-TB patients in a specialist facility for a minimum of six months. In its implementation of the guideline, the South African isolation wards where patients are quarantined in South Africa are described as prison-like by the Southern African AIDS Information Dissemination Service.^19^ To substantiate this fact, Amon et al. highlight that:

[*i*]n March 2009, the AIDS Law Project reported that approximately 1,700 people, including children were detained in TB isolation facilities, many of them in substandard conditions that violated South African constitutional rights and national health legislation.^21^

Eric Goemaere, cited in Amon et al., contends that ‘incarcerating the sick in substandard isolation facilities discourages diagnosis’.^21^ Furthermore, public health experts cited in Amon et al. noted that ‘holding MDR-and XDR-TB patients in overcrowded hospitals with inadequate ventilation increases the risk of nosocomial TB transmissions’.^21^

Furthermore, Doctors without Borders in Cape Town found that more patients would be diagnosed and treated successfully if they were followed treatment in their own homes, rather than being isolated in specialised hospitals.^19^

On the contrary, the WHO recommends that facilities should establish isolation wards,^10^ believing that if these isolation wards are located away from other wards with non-TB patients in a separate building, TB transmission would be prevented.^2^ According to the WHO, ‘two wards housed in separate buildings should be established, namely a medical ward with TB suspects and a TB ward with only patients on TB therapy’.^2^

The WHO further prescribes the following isolation practices that should be enforced strictly during isolation of inpatients:^2^

- ‘Inpatients should not be allowed to leave their rooms or wander the hospital grounds, except for when infectious TB patients must undergo essential diagnostic procedures outside their rooms. Thus, a designated area outside for confirmed infectious TB patients can be used for fresh air’.^2^
- ‘Whenever inpatients leave the isolation areas for medically essential procedures or diagnostic examination, a disposable surgical mask should be given’.^2^
- ‘If possible, visitation hours should be held in a designated area outdoors’.^2^
- Isolation should be continued for a minimum of two weeks except for MDR-TB, which is said to remain infectious for a prolonged period.

The WHO also stipulates that both HCWs and patients must adhere to the airborne precautions in isolation rooms and/or areas.^10^ In addition, settings where isolation facilities do not exist require sophisticated and expensive environmental controls for the reduction of nosocomial transmission. Furthermore, the WHO warns that:

[*t*]he difficulty of ensuring effective separation of patients necessitates the need to avoid hospital admission, or rapidly discharging patients with suspected or confirmed TB, which is sometimes difficult as it is dependent on the seriousness of the patient's condition.^2^

In the light of the possibility that in certain instances it is difficult to discharge patients rapidly or avoid hospitalisation, it is imperative that enough isolation rooms be made available in each hospital.^2^

According to the CDC, ‘the classification of the risk assessment of the health-care setting is used to determine how many isolation rooms each setting needs, depending on the number of TB patients examined’.^23^ The CDC states that:

[*a*]t least one isolation room is needed for settings in which TB patients stay while they are being treated, and additional rooms might be needed depending on the magnitude of patient-days of persons with suspected or confirmed TB disease. Additional rooms might be considered if options are limited for transferring patients with suspected or confirmed TB disease to other settings with isolation rooms.^23^

Furthermore, settings that plan to manage patients with TB disease should have at least one isolation room or enclosure. According to the CDC:

[*i*]solation rooms should be single-patient rooms in which environmental factors and entry of visitors and HCWs are controlled to minimize the transmission of M. tuberculosis. [*Furthermore*], all HCWs who enter an isolation room should wear at least N95 disposable respirators. Visitors may be offered respiratory protection (i.e., N95) and should be instructed by HCWs on the use of the respirator before entering an isolation room.^3^

In addition, isolation rooms should have specific requirements for controlled ventilation, negative pressure, and air filtration. Each patient in the isolation room should have a private bathroom. According to the CDC:

[*p*]ersons with suspected or confirmed TB disease who are in-patients should remain in isolation rooms until they are determined to be non-infectious and have demonstrated a clinical response to a standard multidrug anti-tuberculosis treatment regimen or until an alternative diagnosis is made. Therefore, if the alternative diagnosis cannot be clearly established, even with three negative sputum smear results, empiric treatment of TB disease should strongly be considered.^23^

HCWs should ensure that doors to isolation rooms are kept closed except in cases where patients, HCWs or others need to enter or exit the room.^3^

In addition, respiratory protection with an N95 or higher-level respirator is recommended for personnel entering the TB isolation wards in order to prevent droplet infection of *Mycobacterium tuberculosis*.^20^ Masks must also be placed on coughing patients (cough etiquette/respiratory hygiene) so as to limit potential dissemination of infectious respiratory secretions from the infectious TB patients to others.^20^

### Recommendations

The following simple recommendations were made:

Hospitals should ‘demonstrate a commitment to preventing transmission of infectious agents by incorporating infection control into the objectives of the organization's patient and occupational safety programs’.^21^ Policies and procedures explaining how these measures should be applied, including systems used for the identification and communication of information about patients with potentially transmissible infectious TB, are essential for ensuring the success of these measures.^21^ A key administrative measure that could be taken is the provision of both fiscal and human resources for the maintenance of infection control and occupational health programmes that are responsive to emerging needs. Specific components that could be addressed include facility construction and design decisions,^19^ as well as adequate supplies and equipment, including effective facility ventilation systems.^11^ The involvement of the healthcare administrators in infection control processes would ensure an improvement of their awareness regarding the rationale and resource requirements for the efficient and effective following of recommended infection control practices.

Several administrative factors may affect adherence to isolation practices in healthcare settings namely institutional culture, individual worker behavior, and the work environment.

In their 2005 *Guidelines for preventing the transmission of Mycobacterium tuberculosis in health-care settings*, the CDC state that:

[*s*]afety culture (or safety climate) refers to a work environment where a shared commitment to safety on the part of management and the workforce is understood and followed. A safety culture is created through (1) the actions management takes to improve patient and worker safety; (2) worker participation in safety planning; (3) the availability of appropriate protective equipment; (4) influence of group norms regarding acceptable safety practices; and (5) the organization's socialization process for new personnel. [*Each of these factors was found to have*] a direct bearing on adherence to transmission prevention recommendations.^24^

Education regarding improvements to adherence is one of the IPC interventions that has been studied in detail. It is interesting to note that self-reported adherence is much higher in groups that have received some level of educational intervention.^24^ Educational interventions incorporating video-taped and performance feedback are said to be more successful with regard to improving adherence.^25^

## Conclusion

Isolation practices suggested by the national as well as international legislative frameworks regarding infectious TB patients were not being adhered to in rural hospitals of Vhembe district.

## References

[1] Escombe AR, Moore DAJ, Gilman RH, et al Upper-room ultraviolet light and negative air ionization to prevent tuberculosis transmission. PloS Med. 2009;6(3):e1000043 http://dx.doi.org/10.1371/journal.pmed.1000043 10.1371/journal.pmed.1000043 PMC2656548 19296717

[2] World Health Organization Guidelines for the prevention of tuberculosis in health care facilities in resource-limited settings [document on the Internet]. c1999 [cited 2014 Sep 14]. Available from: http://www.who.int/tb/publications/who_tb_99_269/en/

[3] US Centers for Disease Control and Prevention Guidelines for preventing the transmission of Mycobacterium tuberculosis in health-care facilities, 1994. MMWR. 1994;43(RR-13):1–141. 8602125

[4] Sissolak D, Marais F, Mehtar S. TB infection control prevention and control experiences of South African nurses – a phenomenological study. 2011;11:262. 10.1186/1471-2458-11-262 PMC3096589 21518434

[5] World Health Organization Health care-associated infections fact sheet [document on the Internet]. 2013 [cited 2013 May 14]. Available from: http://www.who.int/gpsc/country-work/gpsc_ccisc_fact_sheet_en.pdf

[6] Lin CF, Yang CY, Lu MS, et al Effectiveness of a nosocomial infection control training in improving knowledge in patient-hired attendants and outsourced workers in Taiwan. J Nurs Res. 2006;16(3):187–194. http://dx.doi.org/10.1097/01.JNR.0000387305.96622.5b 18792888 10.1097/01.jnr.0000387305.96622.5b

[7] World Health Organization Global tuberculosis control 2010 [document on the Internet]. c2010 [cited 2014 Sep 14]. Available from: http://www.who.int/tb/publications/global_report/2010/en/

[8] Tshitangano TG, Maputle MS, Netshikweta ML. Practices of tuberculosis sputum specimen collection at resource-limited hospitals in Vhembe district, Limpopo Province, South Africa. Afr J Phys Health Educ Recr Dance. 2013;19(Suppl 1):116–125.

[9] Tshitangano TG, Maputle MS, Netshikweta ML. Environmental tuberculosis control measures at resource-limited hospitals in Vhembe district, Limpopo Province, South Africa. Afr J Phys Health Educ Recr Dance. 2013;19(Suppl 1):107–115.

[10] World Health Organization WHO policy on TB infection control in health-care facilities, congregate settings and households [document on the Internet]. c2009 [cited 2014 Sep 14]. Available from: http://whqlibdoc.who.int/publications/2009/9789241598323_eng.pdf 24432438

[11] Kumar D. Research methodology: A step-by-step guide for beginners 3rd ed Washington, DC: SAGE Publications Ltd; 2011.

[12] O'Leary Z. The essential guide to doing research. London: SAGE Publications Ltd; 2004.

[13] Tesch R. Qualitative research: analysis types and software tools. New York: Routledge; 1990.

[14] Creswell JW. Research design: qualitative, quantitative, and mixed methods approaches 3rd ed London: SAGE Publications Ltd; 2009.

[15] Lincoln Y, Guba E. Naturalistic inquiry. London: SAGE Publications Ltd; 1985.

[16] Basu S, Andrew J, Poolman EM. Prevention of nosocomial transmission of extensively drug-resistant tuberculosis in rural South African district hospitals: an epidemiological modelling study. Lancet. 2007;370(9597):1500–1507. http://dx.doi.org/10.1016/S0140-6736(07)61636-5 17964351 10.1016/S0140-6736(07)61636-5 PMC3711808

[17] Knirsch CA, Jain N, Pablos-Mendez A, et al Respiratory isolation of tuberculosis patients using clinical guidelines and an automated clinical decision support system. Infect Control Hosp Epidemiol. 2008;19(2):94–100. http://dx.doi.org/10.2307/30141996 9510106 10.1086/647773

[18] US Centers for Disease Control and Prevention Tuberculosis control laws and policies: a handbook for public health and legal practitioners [document on the Internet]. c2009 [cited 2014 Sep 14]. Available from: http://www.cdc.gov/tb/programs/TBLawPolicyHandbook.pdf

[19] Schablon A, Beckmann G, Harling M, et al Prevalence of latent tuberculosis infection among health care workers in a hospital for pulmonary diseases. J Occup Med Toxicol. 2009;4:1 http://dx.doi.org/10.1186/1745-6673-4-1 10.1186/1745-6673-4-1 PMC2631010 19134168

[20] Siegel JD, Rhinehart E, Jackson M, et al Guideline for isolation precautions: preventing transmission of infectious agents in healthcare settings [document on the Internet]. c2007 [cited 2014 Sep 14]. Available from: http://www.cdc.gov/hicpac/pdf/isolation/isolation2007.pdf

[21] Amon JJ, Girard F, Keshavjee S. Limitations on human rights in the context of drug-resistant tuberculosis: A reply to Boggio et al. Health and Human Rights. 2009;11(1):1–10.

[22] Republic of South Africa: Department of Health The national infection prevention and control policy & strategy [document on the Internet]. c2007 [cited 2014 Sep 23]. Available from: http://www0.sun.ac.za/ruralhealth/ukwandahome/rudasaresources2009/DOH/5%20-%20ipc-policy.pdf

[23] US Centers for Disease Control and Prevention Guidelines for environmental infection control in health-care facilities. Recommendations of CDC and the Healthcare Infection Control Practices Advisory Committee (HICPAC). MMWR. 2003;52(RR-10):1–48. 12836624

[24] US Centers for Disease Control and Prevention Guidelines for preventing the transmission of Mycobacterium tuberculosis in health-care settings, 2005. MMWR. 2005;54(RR-17):1–141. 16382216

[25] US Centers for Disease Control and Prevention Tuberculosis transmission in a homeless shelter population – New York, 2000–2003. MMWR. 2005;54(6):149–152. 15716807

